# Move beyond free radical theory of aging

**DOI:** 10.1002/imo2.70079

**Published:** 2026-02-04

**Authors:** Xiaofeng Dai, Meilan Hu, Ruohan Lyu

**Affiliations:** ^1^ National Local Joint Engineering Research Center for Precision Surgery & Regenerative Medicine Shaanxi Provincial Center for Regenerative Medicine and Surgical Engineering, First Affiliated Hospital of Xi′an Jiaotong University Xi′an China; ^2^ Department of Preventive and Health Affiliated Hangzhou First People's Hospital, School of Medicine, Westlake University Hangzhou China; ^3^ Department of Biostatistics, Mailman School of Public Health Columbia University New York USA

**Keywords:** aging, AMP‐activated protein kinase, cold atmospheric plasma, free radical theory, mitochondrial DNA, redox homeostasis

## Abstract

Aging is a gradual pathophysiological process with the loss of functional integrity and gain of disease vulnerability. Mitochondrial DNA is one essential regulatory factor of aging. The canonical free radical theory attributes aging to oxidative damage of mitochondrial DNA caused by free radicals. However, the general validity of this theory has been challenged by incremental evidences, including some seemingly paradoxical reports, which motivates us to substantiate this theory with our current understandings on free radicals and aging. After reviewing this canonical theory and its adapted models, we provided updated insights by emphasizing the dosage‐dependent dual roles of free radicals played during aging and characterizing adenosine 5'‐monophosphate‐activated protein kinase as the nexus between mitochondrial health and free radicals. For practical use, we provided detailed recommendations regarding the dosages of reactive species feasible for treating different health concerns. Additionally, we proposed cold atmospheric plasma (CAP), which is composed of a cocktail of free radicals, as an innovative redox modulatory tool for anti‐aging if used appropriately, alone or in combination with other interventions. Our view not only centers mitochondrial redox disequilibrium in the progression of aging, but also underpins the essential value of the fourth state of matter as represented by CAP in keeping redox homeostasis for the sake of human health that should be appropriately harnessed.

## Highlights

- Emphasize the dose‐dependent dual roles of free radicals on aging.
- Identify adenosine 5'‐monophosphate‐activated protein kinase (AMPK) as the nexus between mitochondria health and free radicals.
- Characterize the dose‐dependent effect of free radicals on AMPK.
- Differentiate doses of free radicals feasible for treating different health concerns.
- Propose cold atmospheric plasma (CAP) as a redox tool for anti‐aging and mechanistic investigations.

## Graphical abstract

Free radicals play a dual, dose‐dependent role in aging. Low levels activate the key sensor adenosine 5'‐monophosphate‐activated protein kinase (AMPK), promoting cell survival mechanisms like autophagy and enhancing mitochondrial health. High levels, however, push AMPK to initiate cell death pathways, such as apoptosis. This positions AMPK as a central nexus linking free radical signaling to mitochondrial function and aging outcomes. Based on this principle, cold atmospheric plasma (CAP), as a controllable source of reactive species including free radicals, offers therapeutic potential. Lower doses can support health and treat degenerative diseases by boosting cellular resilience and survival. Conversely, higher doses can be applied to eliminate malignant cells, making CAP a promising tool against proliferative diseases like cancers.

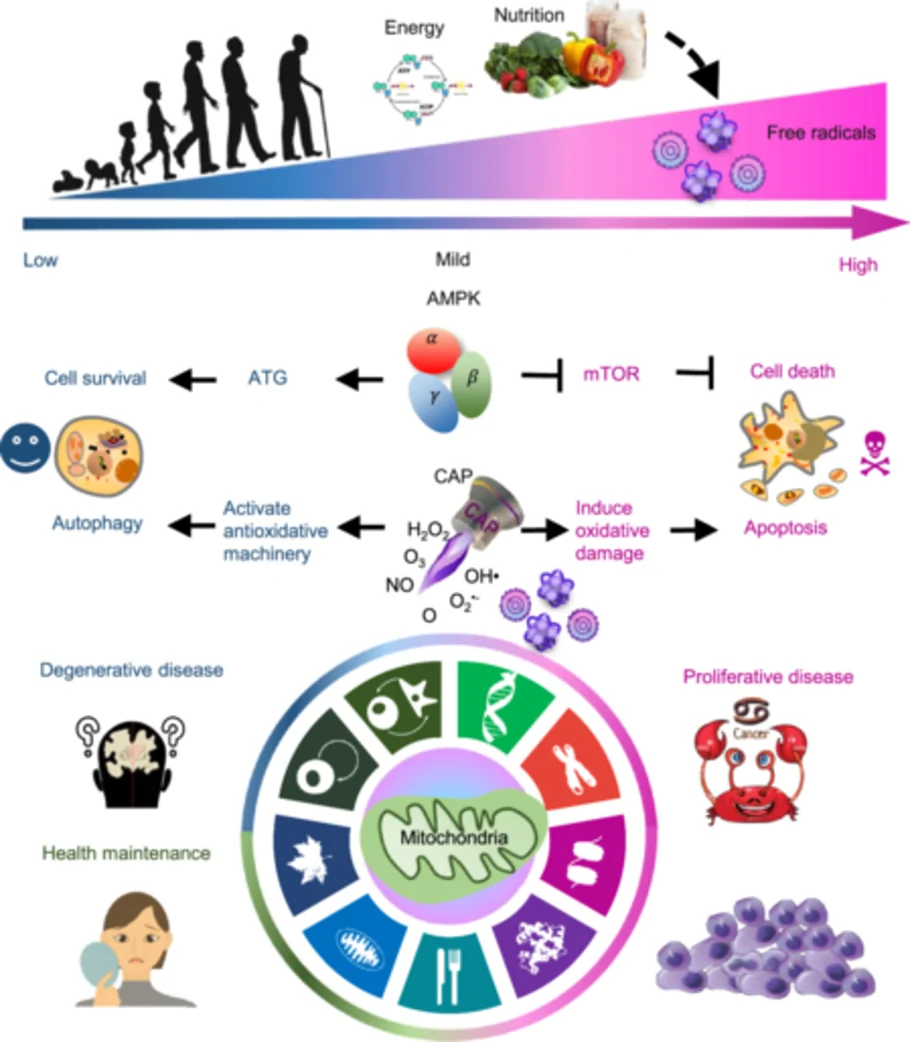

## 1 INTRODUCTION

The lifespan, defined as the duration of the time alive, is a measure of the quantity of life. The healthspan, on the other hand, is an evaluation of the quality of life that refers to the duration of the time an individual can live without the ravages of disease. Aging affects both the quantity and quality of life, and is manifested as the combination of loss of youthful, rejuvenative features and the gain of detrimental, disease‐associated properties. This deterioration process that accompanies aging predisposes individuals to an increased risk of developing major human pathologies, including many degenerative diseases, metabolic syndromes, and cancers [1].

Among the nine hallmarks of aging, that is, genomic instability, telomeric shortening, altered methylation patterns, mitochondrial dysfunction, loss of proteostasis, stem cell exhaustion, cellular senescence, altered intercellular communication, and deregulated nutrient sensing [1], “mitochondrial dysfunction” is a shared property among neurodegenerative diseases (i.e., pathological protein aggregation, synaptic and neuronal network dysfunction, aberrant proteostasis, cytoskeletal abnormalities, altered energy homeostasis, DNA and RNA defects, inflammation, neuronal cell death [2]), metabolic syndromes (defined as a group of risk factors of metabolic origin [3, 4, 5]) and cancers (i.e., sustaining proliferative signaling, evading growth suppressors, resisting cell death, enabling replicative immortality, inducing angiogenesis, activating metastasis, deregulating cellular energetics, avoiding immune destruction, tumor‐promoting inflammation, genome instability [6]). Thus, mitochondria health is centered at the core of human physiological integrity, the progressive loss of which during aging is associated with impaired vitality and increased death vulnerability.

Mitochondria, providing the energy and power of the cell, link electron transport and proton pumping with oxidative phosphorylation (OXPHOS) via a so‐called “electron transport chain” that is leaky during respiration [7]. Mitochondrial DNA (mtDNA) is an important factor participating in the regulation of aging [8, 9, 10]. Free radicals are also referred to as reactive oxygen and nitrogen species (RONS), which include, for example, superoxide anion radical (O_2_^•^^−^), hydroxyl radical (OH•), singlet oxygen (^1^O_2_), and nitric oxide (NO). They are highly reactive atoms or molecules with one or more unpaired electrons that can be formed when molecular oxygen (O_2_) interacts with certain molecules. It is believed that RONS, generated either endogenously by mitochondria or exogenously outside the cell, can initiate the aging process via causing damages to the mitochondria. As our understanding of free radicals and aging has deepened, increasing evidence has emerged to challenge the validity and accuracy of the canonical theory of aging. This has led to the proposal of several adapted versions such as the polymerase γ error model and the spontaneous mtDNA decay model [11, 12] (Figure 1). The contemporary view of aging mechanisms has shifted from a simplistic focus on ROS‐induced oxidative damage as the primary driver to a more integrated paradigm that emphasizes replication error‐driven mtDNA mutagenesis, the hormetic role of ROS in signaling, and the centrality of nutrient‐sensing pathways. This transition can be exemplified by studies on mtDNA mutated mice, which demonstrated that defects in the mitochondrial DNA polymerase proofreading function, leading to an elevated burden of mtDNA mutations and deletions, were sufficient to induce a progeroid phenotype, thereby highlighting replication errors as a critical source of genomic instability [13, 14]. Concurrently, the concept of mitohormesis has been deepened, as evidenced by interventions like methionine restriction, which extended the healthspan not merely by suppressing ROS but also by inducing a compensatory adaptive response that reconfigures mitochondrial bioenergetics and hydrogen sulfide signaling, recasting ROS as signaling molecules in a complex network [15]. Furthermore, the aging field has moved toward pathway‐centric overviews, where master regulators like AMPK and the sirtuin‐NAD^+^ axis have been recognized as key integrators of metabolic and proteostatic signals [16, 17]; this is translationally reflected in clinical trials investigating NAD^+^ precursors for Alzheimer's disease [18], aiming to engage these pathways to improve mitochondrial homeostasis and counteract age‐related decline. While these adapted theories and updated insights mitigated the promotive effect of free radicals in aging, balanced views on free radicals in human health under both physiological and pathological conditions are needed, especially in the context of aging. In response, we emphasize the context‐dependent dual role of AMPK across different disease spectra, propose a unifying “degenerative versus proliferative disease × free radical dose” intervention framework, and incorporate cold atmospheric plasma (CAP), a multimodal redox editing tool, into this framework accordingly in a systematic way.

**FIGURE 1:**
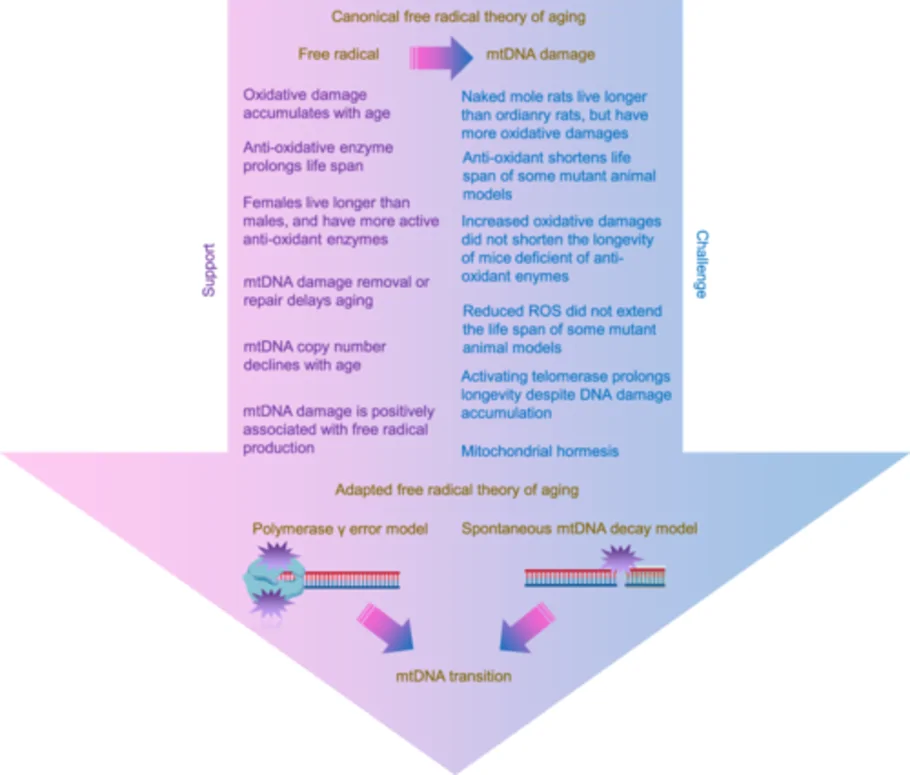
Summarized illustration on the current status of the free radical theory of aging. The canonical free radical theory of aging primarily attributes aging to free radical‐caused mitochondria DNA (mtDNA) damage. Evidence supporting this theory includes, for example, oxidative damage accumulates with age, anti‐oxidative enzyme prolongs lifespan, females live longer than males, who express estrogens that have promotive roles on the activity of anti‐oxidant enzymes, mtDNA damage removal or repair delays aging, mtDNA copy number declines with age, and mtDNA damage is positively associated with free radical production. However, this theory has been challenged by many evidence including, for example, naked mole rats live longer than ordinary rats but have more oxidative damages, anti‐oxidant shortens the lifespan of some mutant animal models, increased oxidative damages did not shorten the longevity of mice deficient of anti‐oxidant enzymes, reduced reactive oxygen species (ROS) did not extend the lifespan of some mutant animal models, activating telomerase prolongs the longevity despite DNA damage accumulation, and the recognition of mitochondrial hormesis. This has led to the establishment of adapted free radical theories of aging that attribute aging to mtDNA transition instead of free radicals but by different causes. There currently exist two adapted models, that is, polymerase γ error theory and the spontaneous mtDNA decay theory, which consider defects in the mitochondrial polymerase γ (a vital enzyme responsible for mtDNA replication) and spontaneous mtDNA decay as the two primary sources of mtDNA transition.

By reviewing the central dogma of “The Free Radical Theory of Aging” and its adapted versions in Section 2, we proposed our updated insights to substantiate the content of this canonical theory in Section 3, which emphasized the dose‐dependent dual functionalities of free radicals in human health and the central role of adenosine 5'‐monophosphate‐activated protein kinase (AMPK) in sensing internal or external free radicals for adapting cells to the nutrient and energy conditions to keep the mitochondria health. Based on these insights, we summarized the principles of using approaches based on free radicals for health management in Section 4, and proposed CAP as a feasible approach for treating syndromes associated with advanced age in Section 5 prior to the concluding remarks.

## 2 CANONICAL FREE RADICAL THEORY OF AGING

### 2.1 Canonical theory

The traditional free radical theory stated that phenotypes associated with aging can be attributed to accumulated oxidative damage to macromolecules by endogenous or exogenous RONS [19] (Figure 2). Several enzymes, such as nicotinamide adenine dinucleotide phosphate (NADPH) oxidase (NOX), superoxide dismutase (SOD), and nitric oxide synthase (NOS), are primarily involved in endogenous RONS production [20]. RONS can be produced from multiple cellular compartments, where mitochondria are the largest source of endogenous RONS such as H_2_O_2_, O_2_^•^^−^, OH•, and •NO [21]. In short, NOX converts ^1^O_2_ to radical O_2_^•^^−^ that dismutates to H_2_O_2_ by SOD and further reacts to form OH• through the Fenton reaction [20]. NOS, with three isoforms (i.e., epithelial NOS, neuronal NOS, and inducible NOS), catalyzes l‐arginine to •NO [22] that reacts with O_2_^•^^−^ to form peroxynitrite (ONOO^−^) [23] (Figure 3). In mitochondria, the outer membrane enzyme monoamine oxidase provides a large source of H_2_O_2_ that further enhances the concentrations of free radicals within both the mitochondrial matrix and cytosol via catalyzing the oxidative deamination of biogenic amines [20]. Exogenous RONS can interact with cell surface receptors to generate endogenous oxidative species or penetrate through membrane channels such as aquaporin family members (AQPs) (Figure 3). Exogenous sources of RONS include, for example, nutrient supply such as vitamins, cofactors, polyphenols; certain life styles such as physical exercises, intermittent fasting, and mild hypoxia; toxic intake such as tobacco, alcohol and smoky food; environmental pollution such as air/water pollutants, heavy metals such as cadmium (Cd), mercury (Hg), lead (Pb), iron (Fe) and Arsenic (As); drugs such as cyclosporine, tacrolimus, gentamycin, and bleomycin [20, 24]; and therapeutic interventions such as radiation, photodynamic therapy, and photothermal therapy (Figure 4).

**FIGURE 2:**
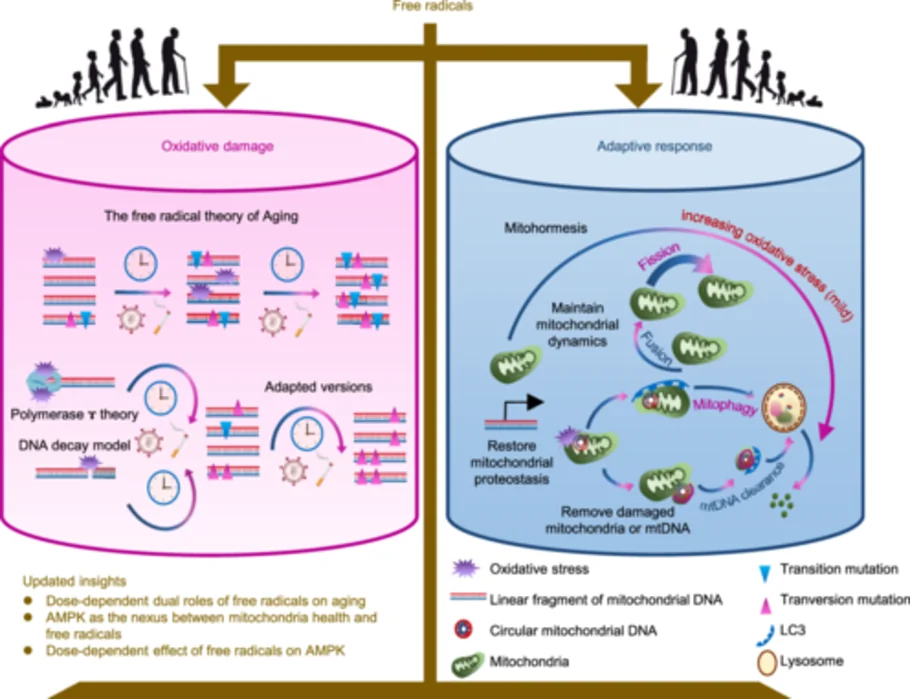
The canonical free radical theory of aging, its adapted versions, and updated insights. The free radical theory of aging states that aging is primarily caused by accumulated oxidative damage to the mitochondrial DNA (mtDNA). Recent studies have found that it is transition mtDNA mutations rather than transversion mutations that gradually accumulate overtime and reach the pathological levels via clonal expansion. Based on this finding, one adaptation of the free radical theory of aging is the polymerase ɤ theory of aging, which states that transition mutations are caused by replication errors made by the disrupted fidelity of mitochondrial polymerase ɤ as a result of its interactions with free radicals. An alternative model is the spontaneous decay model that attributes the incremental somatic transition mutations to the spontaneous decay of mtDNA. Conditions capable of accelerating aging via oxidative damage include, for example, chronic virus infections and behaviors causing chronic inflammation, such as tobacco smoking. In addition, mitohormesis has been proposed to explain the observed beneficial role of free radicals for maintaining mitochondria health, where mild oxidative stress can restore mitochondria proteostasis, maintain mitochondrial dynamics and remove damaged mitochondria (mitophagy) or mtDNA clearance with the increased dosage. The updated insights proposed here can be summarized into three points: **①**Dose‐dependent dual roles of free radicals on aging (high dose of free radicals can accelerate aging via creating oxidative damage to mitochondria, low dose of free radicals can activate the anti‐oxidative machinery to restore mitochondria health); **②**AMP‐activated protein kinase (AMPK) as the nexus between mitochondria health and free radicals; **③**Dose‐dependent effect of free radicals on AMPK.

**FIGURE 3:**
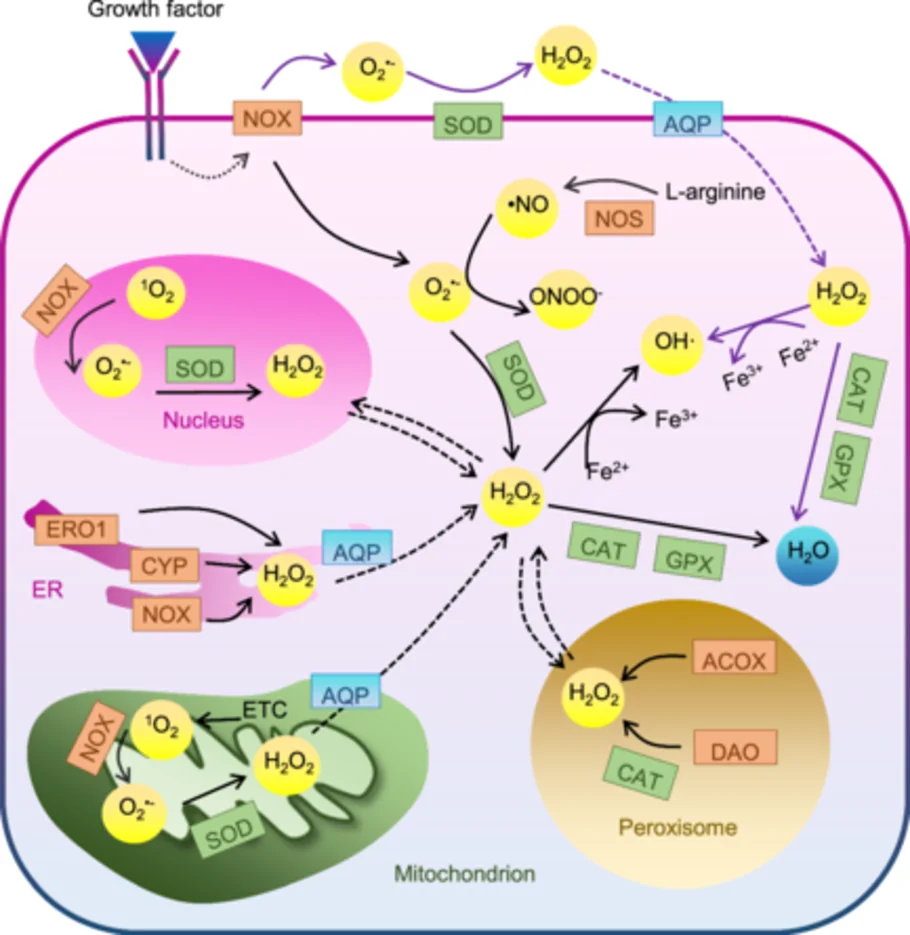
Simplified illustration on primary sources of endogenous reactive oxygen and nitrogen species (RONS). Reactive oxygen species and other active species are generated via various endogenous sources that are distributed in different cellular compartments including the cell membrane. Nicotinamide adenine dinucleotide phosphate (NADPH) oxidase (NOX) converts ^1^O_2_ to O_2_^•^^−^ that dismutates to H_2_O_2_ by superoxide dismutase (SOD) and further reacts to form OH• through the Fenton reaction. Nitric oxide synthase (NOS) catalyzes l‐arginine to •NO that reacts with O_2_^•^^−^ to form peroxynitrite (ONOO^−^). Exogenous RONS can interact with cell surface receptors to generate endogenous oxidative species or penetrate through aquaporin (AQP). H_2_O_2_ can be further converted to H_2_O by enzymes such as catalase (CAT) or glutathione peroxidase (GPX). ACOX, acyl‐CoA oxidase; AQP, aquaporin; CAT, catalase; CYP, cytochrome P450‐dependent monooxygenases; DAO, d‐amino acid oxidase; ER, endoplasmic reticulum; ERO1, endoplasmic reticulum oxidoreductin 1; ETC, electron transport chain; GPX4, glutathione peroxidase 4; NOS, nitric oxide synthase; NOX, NADPH oxidase; SOD, superoxide dismutase; UCP, uncoupling protein. Solid arrows denote species conversion or generation; dashed arrows represent spatial translocation; and densely dotted arrows signify indirect regulation. Black arrows depict intracellular radical generation, whereas purple arrows trace the extracellular generation and penetration of H_2_O_2_, along with its subsequent conversion to other radicals.

**FIGURE 4:**
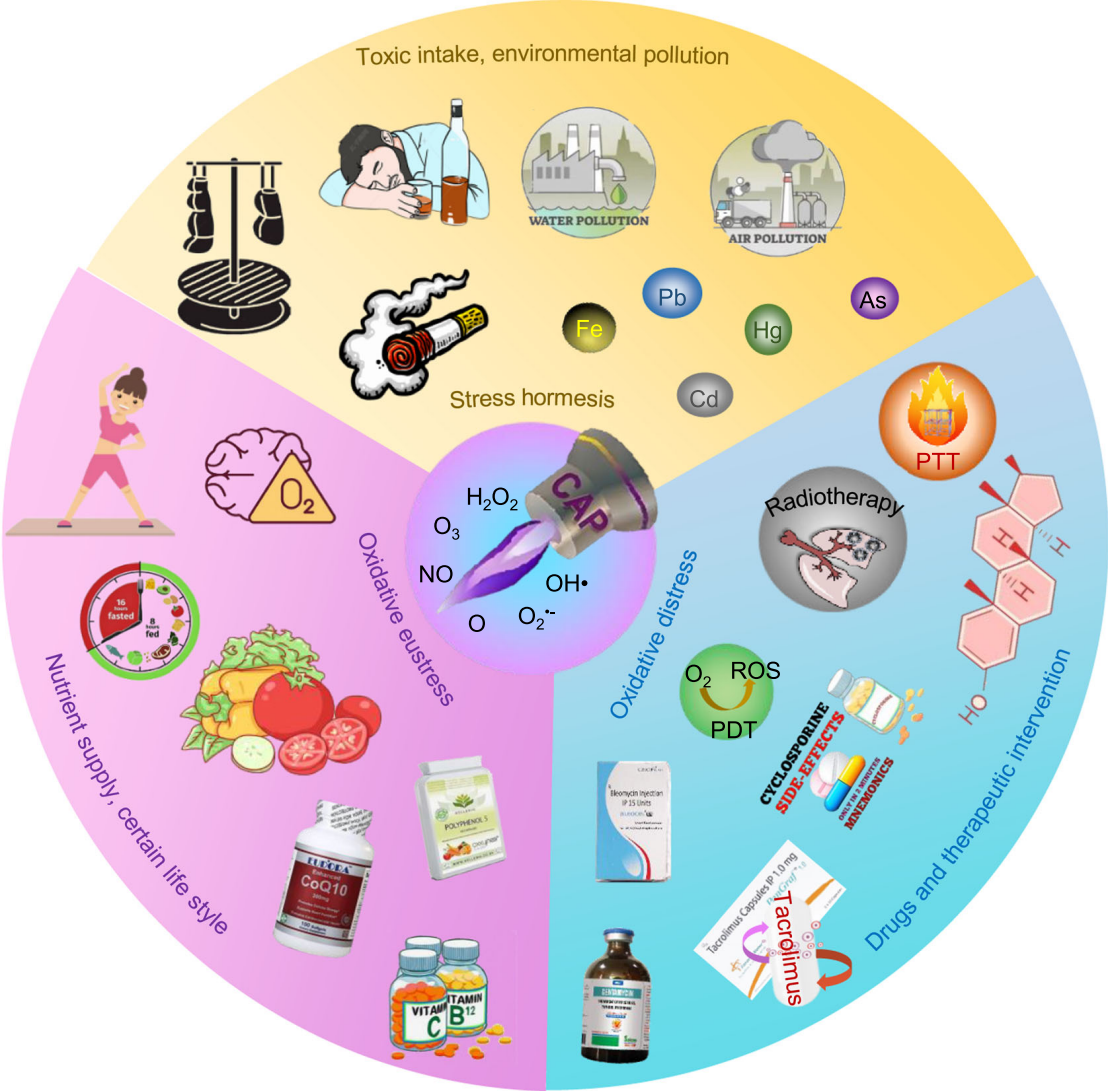
Simplified illustration on primary sources of exogenous RONS. The totality of exposures to RONS cover diverse aspects of life and can exert oxidative eustress, stress hormesis, or oxidative distress depending on exposure intensity. Nutrient supply, such as vitamins, cofactors, polyphenols, and certain lifestyles, such as physical exercises, intermittent fasting, and mild hypoxia convey physiological levels of oxidants that support physiology (i.e., oxidative eustress). Toxic intake, such as tobacco, alcohol, smoky food, and environmental pollutants such as various heavy metals may cause adaptation to the stress and resilience (i.e., stress hormesis); drugs and therapeutic interventions, such as radiotherapy, photodynamic therapy, and photothermal therapy take actions while imposing excessive oxidant exposure (i.e., oxidative distress). Cold atmospheric plasma (CAP), being a dose‐dependent redox modulatory tool, can be potentially employed as a redox medicine under both physiological and pathological conditions by maintaining redox homeostasis. PDT, photodynamic therapy; PTT, photothermal therapy.

The free radical theory of aging emphasizes the driving role of accumulative oxidative cellular damage in aging and attributes such detrimental effects to free radical‐caused mitochondrial DNA (mtDNA) mutations [25]. It is known that the electron transport chain is a primary source of free radicals, and mtDNAs are located in the proximity of the electron transport chain. Thus, it is postulated that mtDNA mutations would promote mitochondrial dysfunction and form a mutual promotive vicious loop towards enhanced free radical production and mtDNA mutation.

One milestone evidence supporting this theory was the observation of oxidative damage in the form of 7,8‐dihydro‐8‐oxo‐deoxyguanosine (8‐oxodG) DNA oxidative lesions that accumulated with age [26]. Another study has correlated longevity with the oxidant production rate by analyzing two animal species sharing similar size and oxygen consumption rates but divergent lifespans, that is, rats (3–4 years) and pigeons (35 years), where the authors amazingly found that the oxidant production rate by mitochondria of rats was over 30% higher than that of pigeons [27]. On the other hand, one study has established a positive linear correlation between the activity of the antioxidant SOD and the lifespan by examining various species ranging from mice to monkeys, chimpanzees, and humans [28], and the observation on the promotive role of anti‐oxidative enzyme (including SOD) over‐representation in prolonging the lifespan of *Drosophila* was a critical impetus to this line of work [29]. Similarly, it has been documented that over‐expressing *p53* (a gene encoding a well‐known tumor suppressor) significantly enhanced the animal longevity independent of its tumor suppressive role [30], given the activating role of p53 on multiple antioxidant enzymes [31]. Also, it has been observed that females live, in general, longer than males due to the stimulative role of estrogens on the expression of anti‐oxidative enzymes such as glutathione peroxidase (GPX) and manganese superoxide dismutase (MnSOD) [32]. Consistent with this, females produced fewer free radicals and enjoyed longer longevity than males who produced more free radicals and lived shorter lifespan [33, 34].

Supportive evidence has also been identified from mtDNA. For example, mtDNA is a high copy number genome required for OXPHOS; the copy number of mtDNA declined linearly with age, and was associated with heteroplasmic single‐nucleotide variants that accumulated sharply after the age of 70 [35]. MTFP1 (mitochondrial fission process 1) was identified as one key protein responsible for the decreased mtDNA copy number with advanced age, which maintains the quality of inner mitochondrial membrane (IMM) by removing damaged IMM subdomains [36]. Lysosomes in proximity to the mitochondria engulfed the herniating IMM, the process of which was enhanced in the mitochondria undergoing oxidative stress [37]. TFAM (mitochondrial transcription factor A) was found to help eliminate leaked mtDNA by interacting with the autophagy protein LC3 (microtubule‐associated protein 1A/1B‐light chain 3), linking mtDNA damage with inflammation and aging [38]. A recent study reported that the functional longevity of cells could be prolonged by ATFS‐1 (activating transcription factor associated with stress‐1) that counteracted age‐dependent mtDNA damage by promoting repair over transcription [39]. On the other hand, clues on the genetic connections between mtDNA damage and abnormal free radical production were found. Mitochondrial OXPHOS complexes are assembled from proteins encoded by both mtDNA and nuclear DNA; and several genes coordinating gene expression across organelles such as NDPK (uncharacterized nucleoside diphosphate kinase) were found to control OXPHOS [40]. In addition, 14 proteins encoded by mtDNA have been identified to be essential in OXPHOS so far, with CYTB‐187AA (cytochrome b − 187 amino acid) being the most recently discovered [41].

### 2.2 Challenges to the canonical theory

Accumulated evidence has suggested that the canonical free radical theory of aging is single‐sided or half‐completed despite its great popularity in the field of aging. One interesting observation that challenges the integrity of the canonical free radical theory of aging is the much longer lifespans of naked mole rats (i.e., can reach as long as 25 years) than that of ordinary rats (i.e., typically 3–4 years), and higher oxidative damages detected in the naked mole rats than that in the ordinary rats [42]. In addition, several mutants of *Caenorhabditis elegan*, such as NUO‐6 displayed higher oxidative stress but lived longer than the wildtype controls, the lifespan of which was, however, shortened once being subjected to the treatment of anti‐oxidants such as N‐acetyl cysteine [43]. Similar results have been reported in the *Mclk1* (a gene encoding a mitochondrial enzyme) homozygous mutant mice [44].

Though some studies have attributed, at least partially, the longer lifespans of female as compared with males to the higher activities of antioxidant enzymes such as SOD in response to estrogen, the longevity of mice deficient in both GPX and MnSOD was not decreased despite the increased oxidative damage [45]. Also, there was a lack of correlation between mitochondrial reactive oxygen species (ROS) and the lifespan of *Drosophila*, as *Drosophila* over‐expressing adenine nucleotide translocase (ANT) showed significantly reduced ROS production but no lifespan extension [46]. These studies have unanimously suggested that mitochondrial free radical production was not correlated with the lifespan.

There also exists evidence that the lifespan can be extended without affecting the oxidative damage to cells, further challenging the general validity of the canonical free radical theory of aging. This is achieved by over‐expressing telomerase (the enzyme responsible for maintaining the length of telomeres, and cells lacking telomerase have a replicative limit) together with p53 and p16 in mice, where triple transgenic animals showed around 35% increased average lifespan with statistical significance, suggesting the role of activating telomerase in prolonging the longevity of individuals despite DNA‐damage accumulation [47].

On the other hand, certain levels of free radicals are beneficial and vital in maintaining the normal functionalities of mitochondria. For instance, increased ROS production within the electron transport chain was characterized with an important role in relaying signals indispensable for maintaining the normal functions of mitochondria in *Drosophila* [48]; and low levels of oxidative stress in mitochondria was protective and beneficial for the longevity of *C. elegans* [49]. These findings have led to the concept of mitochondrial hormesis or mitohormesis, where mild oxidative stress could provoke the longevity and metabolic health by stimulating the stress defense in mitochondria [50] (Figure 2).

### 2.3 Adapted free radical theory of aging

Incremental efforts have been devoted to explain the paradoxical observations regarding the outcomes of free radicals on aging, with different hypotheses or models having evolved to update or substantiate the canonical free radical theory of aging [25].

Detailed investigations revealed that mtDNA mutations accumulated with aging did not increase in mtDNA transversion (i.e., A ↔ C, A ↔ T, G ↔ C, G ↔ T) but rather mtDNA transition mutations (i.e., A ↔ G, C ↔ T), the latter of which was known to be caused by mitochondrial polymerase γ errors instead of oxidative damage [11]. It has also been reported that the abundance of mtDNA transition mutations was over 30‐fold higher than that of transversion mutations [51]. These have collectively suggested that aging is not a result of accumulation of free‐radical‐induced oxidative damage but rather an outcome of incremental mtDNA transitions.

Accordingly, one theory has evolved that attributed the mtDNA mutation burden to mtDNA replication errors, namely the polymerase γ theory of aging [11] (Figure 2). Mitochondrial polymerase γ is the vital enzyme responsible for mtDNA replication in mammalian cells, defects of which may substantially endanger DNA replication fidelity. Accumulated mtDNA replication errors over time, once exceeding the tolerance threshold, may lead to a plethora of syndromes associated with advanced age, some of which are pathological. As a cornerstone experiment supporting the vital role of polymerase γ errors during aging, Trifunovic et al. established mice carrying mitochondrial polymerase γ deficient of the proof‐reading functionality that led to a large spectrum of transition mutations in the mtDNAs, premature onset of aging, and a reduced lifespan without elevated levels of oxidative stress [52]. Consistent with this, Sturgis et al. found that polymerase γ exonuclease‐deficient mice displayed enhanced rate of mtDNA mutation, impaired mitochondria function and accelerated aging [53]. Similar results have also been documented in humans, supporting the vital role of mitochondrial polymerase γ rather than oxidative damage in aging [54]. In addition, Itsara et al. expanded the essential role of polymerase γ errors in aging to other organisms using *Drosophila* as the modeling organism, and showed that both the loss‐of‐function of antioxidants and deficient DNA damage repair enzymes could compromise somatic mtDNA integrity [55]. Additionally, oxidative stress was considered to cause mtDNA deletions instead of point mutations including transitions [12]. An alternate model of the polymerase γ theory attributed the positive association between the incremental somatic transition mutations and aging to the spontaneous decay of mtDNA rather than accumulated polymerase γ errors [11]. That is, the accumulation of transition mutations was accelerated during mtDNA replication when the DNA was in a single stranded form and subjected to the spontaneous decomposition of DNA bases [11] (Figure 2). By establishing a causative association between mtDNA transitions and aging, the polymerase γ theory of aging and its alternative model confined the contributions of free radicals to a less significant role. This has revolutionized our conventional concept on aging and motivated us to reexamine the role of free radicals in aging.

## 3 UPDATED INSIGHTS ON THE FREE RADICAL THEORY OF AGING

We propose updated insights on the free radical theory of aging in two aspects. First, we emphasize the dual roles played by free radicals in aging. Second, we propose the pivotal role of AMPK in dictating cell behavior in response to free radicals in a dose‐dependent manner (Figure 5). Though high levels of RONS can damage protein and cause DNA mutation that is linked to various diseases associated with advanced age such as cardiovascular diseases [56], diabetes [57] and cancers [58, 59], low levels of RONS may slow down aging [60] via reacting with phospholipids and cell surface receptors to initiate intracellular signaling and acting as signal transducers [61], where AMPK may function as an important energy sensor to be regulated for the benefits of human health.

**FIGURE 5:**
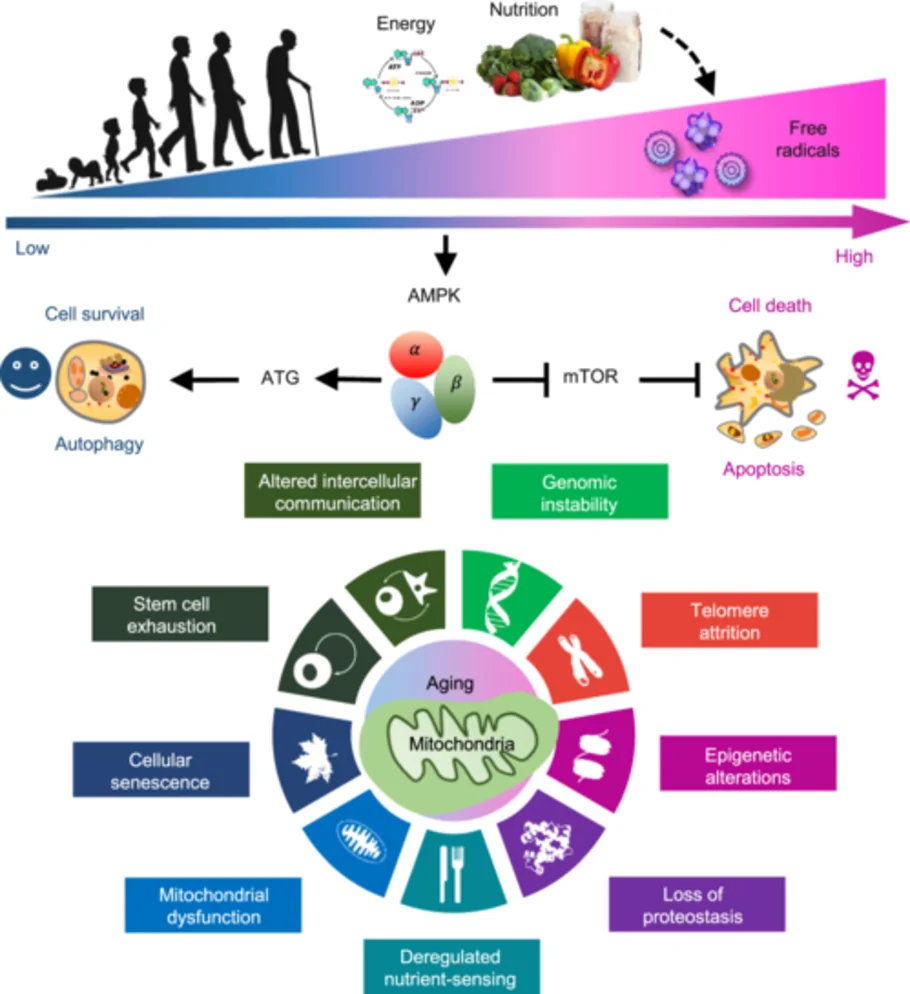
Updated insights on the free radical theory of aging. There are three primary bullets in the proposed insights: **①** Dose‐dependent dual roles of free radicals on aging. Besides causing cell damage, free radicals can also help cell survival on appropriate dosage. **②** AMPK as the nexus between mitochondria health and free radicals. In response to free radical‐induced cellular stress at different levels, AMPK (composed of three sub‐units) can either induce cell death via suppressing mammalian target of rapamycin (mTOR) signaling or trigger cell survival via activating the autophagic pathway, among other downstream players. **③** Dose‐dependent effect of free radicals on AMPK. In general, high level of free radicals may switch AMPK on the path towards cell death, and low level of free radicals may turn on AMPK for cell survival. In summary, aging, as being characterized by 9 hallmarks (i.e., mitochondrial dysfunction, deregulated nutrient‐sensing, loss of proteostasis, epigenetic alterations, telomere attrition, genome instability, altered intercellular communication, stem cell exhaustion, cellular senescence), is a deterioration process of mitochondria that can be modulated via free radicals in a dose‐dependent manner with AMPK being the primary sensor. ATG, autophagy‐related gene; AMPK, adenosine 5'‐monophosphate‐activated protein kinase; mTOR, mammalian target of rapamycin.

Based on these updated insights, we classify common age‐associated disorders into degenerative or proliferative diseases when aging progresses into the pathological state, and differentiate the intervention approaches and driving molecular mechanisms accordingly (Figure 5). Common age‐associated degenerative diseases include, for example, neurodegenerative disorders such as Alzheimer's disease, spinal degenerative diseases such as osteoporosis and intervertebral disc degeneration (IDD). One typical proliferative disease related to aging is cancer, which is characteristic of uncontrolled proliferation. Metabolic syndromes such as type II diabetes are discussed together with degenerative diseases due to their high associations [62, 63].

### 3.1 Free radicals play dual roles in aging in a dose‐dependent fashion

Free radicals play dual roles in aging, and the outcome of cells in response to free radicals highly depends on the magnitude and duration of exposure to the redox stimuli. While high doses of RONS are cell‐toxic and associated with various pathological conditions, low concentrations of RONS are indispensable in intracellular signaling and slight redox stress (either reductive or oxidative) may activate the compensatory mechanisms for retarding aging [20, 21]. As cells at the pathological states typically have higher redox levels than those at the normal states given the elevated cell chaosity during disease initiation and progression (e.g., cancer cells exhibit significantly higher basal levels of free radicals than normal cells [61]), increasing the redox level of pathological cells may trigger the death programs of cells once the perturbation magnitude exceeds the death threshold, and drastically reducing the RONS levels of transformed cells may help retard the progression of transformed cells. On the other hand, reducing the RONS levels of normal cells may prevent them from developing into the pathological states as a result of oxidative damage, and slightly enhancing the RONS levels of healthy cells may activate the anti‐oxidative machinery for health maintenance [20, 21]. In brief, anti‐oxidation (either through direct anti‐oxidative approaches or mild pro‐oxidation for stimulated endogenous anti‐oxidative ability) is, in general, beneficial to healthy individuals for preserving youth and patients carrying degenerative diseases; pro‐oxidation, on the other hand, may be useful to treat diseases with proliferative features for reversing detrimental properties. Distinct types of cells and organelles confer differential sensitivities to free radicals, given their different basal redox states. For instance, the overall redox environment of the nucleus is more reductive than the cytosol for the sake of protecting DNA replication from oxidative damage, and mitochondria have a higher redox level than the cytosol, as it is the primary intracellular source of oxidants such as H_2_O_2_ and O_2_^•−^ [21]. Of note is the importance of the mitochondrial redox metabolism under physiological conditions that spans numerous fundamental aspects. These include, for example, the trigger of programmed cell death in response to abnormal stress, regulation on the morphology and dynamics of mitochondria, and removal of dysfunctional mitochondria. The effect of oxidants on cells and organelles is dose‐dependent. Take mitochondria as an example, while low levels of oxidants induce mitophagy [64, 65], high levels of oxidants may result in macroautophagy [66, 67] or cause cell death through opening the mitochondrial permeability transition pore and releasing additional ROS [68, 69, 70].

Focusing on aging, accumulating evidence has suggested the role of oxidants in extending the lifespan [71, 72], where mitohormesis (i.e., oxidant signaling in mitochondria) in response to stresses such as caloric restriction, mild hypoxia, and physical activity was considered to be essential [60]. Additionally, it has been revealed that early‐life exposure to oxidants extended the lifespan of *Caenorhabditis elegans* due to H_2_O_2_‐induced global reduction of histone 3 Lys4 (H3K4) tri‐methylation [73].

### 3.2 AMPK acts as the nexus between mitochondrial health and free radicals

AMPK plays an essential role in aging that orchestrates an integrated network involving, e.g., forkhead box O (FOXO), mammalian target of rapamycin (mTOR) and NAD‐dependent deacetylase sirtuin (SIRT) [74, 75, 76, 77, 78]. AMPK is an evolutionarily conserved energy sensor with critical roles in regulating redox homeostasis. Specifically, ATP depletion as a result of energy deficiency activates AMPK signaling that, in turn, stimulates catabolic processes and, accordingly, shuts down many anabolic processes as well as several other energy‐consuming activities [79, 80, 81]. AMPK activation is associated with many pathways slowing down aging, for example, the suppressed mTOR pathway, stimulated SIRT signaling, inhibited inflammation, and prevented mitochondrial malfunction. The responsiveness of AMPK declines with aging, which impairs the maintenance of redox homeostasis and, consequently, human longevity and healthgevity. Specifically, aging disturbs the homeostasis of tissues by predisposing them to, for example, the redox stress, resulting in reduced sensitivity to external or internal insults. Thus, young tissues display higher AMPK sensitivities than old tissues to the same stimuli. Accordingly, AMPK activators, including pharmaceutical agents like metformin and therapeutic strategies that counteract AMPK‐suppressing pathways such as the insulin signaling axis, have been increasingly regarded as promising pharmacological interventions for targeting the aging process [82, 83, 84, 85].

#### 3.2.1 Activating AMPK for health maintenance

AMPK activation is beneficial to human health as it can stimulate energy production in response to stress and inhibit energy consumption for the synthesis of proteins, cholesterols and glycogens [86, 87, 88, 89, 90]. Besides maintaining energy metabolism, AMPK activation also induces autophagy to remove damaged structures [91, 92] and stimulates the anti‐oxidative machinery to alleviate redox stress [93], endoplasmic reticulum stress [94, 95], and attenuate inflammation [93, 96, 97]. Interestingly, the oxidative stress and endoplasmic stress are augmented, the autophagic capacity is impaired, and a low level of inflammation develops with the progress of aging. Besides, the AMPK and insulin pathways are mutually inhibitory, that is, many downstream effectors of AMPK, such as FOXO can be suppressed by insulin signaling and *vice versa* [93]. In addition, a large signaling network of transcription factors targeting functional hallmarks of aging such as mTOR, SIRT1, NF‐E2 related factor 1 (NRF1), p53, cyclic AMP response‐binding protein (CREB) are also coordinated by AMPK [98, 99]. Therefore, activating AMPK seems to be favorable for health maintenance and retarding aging.

Lifestyles such as caloric restriction and moderate physical exercises have been recommended for keeping body fitness due to their known roles in stimulating AMPK activity and preventing insulin resistance [100]. Therapeutically, molecules capable of activating AMPK such as metformin, statins, and phytochemicals (e.g., berberine, quercetin, resveratrol) have been considered useful in health maintenance, as well as the prevention and therapy of metabolic and neurodegenerative diseases [76, 101, 102, 103, 104, 105].

#### 3.2.2 Activating AMPK for treating degenerative diseases

Neurodegenerative diseases, including Alzheimer's, Parkinson's, Huntington's, and amyotrophic lateral sclerosis are characterized by the progressive degeneration of nerve cells that eventually leads to dementia [106]. Being the most common neurodegenerative disorder, the pathogenesis of Alzheimer's disease is complicated that involves multiple factors such as autophagy deficiency, mitochondrion failure, insulin resistance, and oxidative stress [107, 108]. The role of AMPK in Alzheimer's disease seems to be controversial as, on one hand, AMPK activation is capable of inducing cell autophagy, promoting mitochondrial synthesis, and enhancing insulin sensitivity; yet, on the other hand, AMPK phosphorylation functions as a tau kinase in response to metabolic stresses such as amyloid beta (Aβ) peptides exposure [109, 110]. However, phosphorylated AMPK did not co‐aggregate with tau in tangles, and AMPK phosphorylation at Ser485 was an inhibitory AMPK phosphorylation site, prohibiting it from being further phosphorylated at Thr172 (a critical site residing in the catalytic core of AMPK [111]). Thus, it is necessary to focus on the phosphorylation profile of AMPK during the pathogenesis of Alzheimer's diseases, and AMPK agonists may represent a promising approach for treating Alzheimer's diseases once the exact mechanism has been clarified. Indeed, AMPK activation induced by leptin or metformin reduced tau phosphorylation and consequently attenuated Alzheimer's disease progression [102].

AMPK has been shown to be protective against IDD via inducing autophagy. By up‐regulating the ratio of B cell lymphoma 2 (Bcl‐2) and Bcl‐2‐associated X protein (Bax) and down‐regulating the level of cleaved caspase‐3, AMPK‐induced autophagy rescued rat nucleus pulposus cells from undergoing apoptosis in response to tert‐Butyl hydroperoxide (TBHP) exposure [112]. Also, by increasing the expression of p21WAF1 (p21, which is a cell cycle checkpoint effector and inducer of senescence) and p16INKa (p16, which is an inhibitor of CDK4), and up‐regulating the phosphorylation of p53, AMPK‐mediated autophagy suppressed TBHP‐induced senescence of rat nucleus pulposus cells; by modulating the expression of anabolic genes such as *Col2A1* (a gene encoding the pro‐alpha 1 chain of type II collagen) and *Acan* (a gene encoding aggrecan i.e. a multimodular proteoglycan), as well as catabolic genes such as *MMP3/13* (genes encoding matrix metalloproteinase 3 and 13) and *ADAMTS4/5* (genes encoding ADAM metallopeptidase with thrombospondin type I motif 4 and 5), AMPK‐activated autophagy avoided TBHP‐triggered extracellular (ECM) destruction [113]. Several crucial proteins, such as mTOR and SIRTs play pivotal roles in the regulation of AMPK and autophagy during IDD progression. Specifically, activated AMPK significantly reduced mTOR phosphorylation in human nucleus pulposus cells exposed to TBHP; inactivated mTOR led to autophagosome assembly via activating UNC‐51‐like kinase 1 (ULK1) [113]. On the other hand, AMPK‐induced autophagy was accompanied with up‐regulated expression of SIRT3 for suppressed mitochondrial apoptosis and senescence in response to TBHP treatment [114], inhibited secretion of pro‐inflammatory cytokines, and enhanced ECM homeostasis stimulated by oxidative damage in rat nucleus pulposus cells [115]. Besides, AMPK protected human nucleus pulposus cells from advanced glycan end‐product (AGE)‐induced mitochondrial apoptosis by increasing SIRT3 expression [116]. It is also worth noting that, despite the overall beneficial role of AMPK activation in IDD, the deleterious effect of AMPK on such diseases has also been reported. Instead of inducing autophagy, AMPK activation triggered cell death instead of autophagy for cell survival in these reported cases. Specifically, inhibited mTOR as a result of AMPK activation accelerated the apoptosis and senescence of degenerative rat nucleus pulposus cells [117] and degenerative human annulus fibrosus cells [118].

Given the vital role of AMPK in degenerative disorders, therapeutic strategies activating AMPK have been proposed for treating such diseases. For instance, metformin, a known AMPK agonist, capable of inducing autophagy [119] and protecting degenerative cells from apoptosis, senescence and ECM degradation [120, 121], has been shown effective in ameliorating many degenerative syndromes such as neurodegenerative diseases [122] and disc degenerative disorders [123], and has become the first‐line medicine for treating type II diabetes. Akin to metformin, aspirin is protective of cells from lipopolysaccharide‐induced oxidative injury via activating AMPK signaling pathway [124], the therapeutic effect of which indicates innovative use of conventional drugs in treating degenerative diseases such as IDD. It is worth mentioning that drugs activating AMPK have also been shown effective in treating metabolic syndromes such as type II diabetes, alcoholic liver, cardiovascular diseases, and obesity [125, 126, 127, 128, 129, 130], given the predisposing role of metabolic disorders in degenerative diseases [62, 131].

#### 3.2.3 Inhibiting AMPK for treating proliferative diseases

Being an aging disease characteristic of uncontrolled proliferation [6, 132], accumulated evidence has suggested the oncogenic role of AMPK during cancer progression via facilitating cancer cell survival [133]. For instance, AMPK‐deficient cells were resistant to oncogenic transformation and tumorigenesis [134, 135]; AMPK promoted the survival of breast cancer cells [136] and T cell acute lymphoblastic leukemia cells [137], the efficacy of which may be attributed to its role in switching cell metabolism from favoring aerobic glycolysis to mitochondrial oxidative respiration for mitigated metabolic stress [138]; and AMPK activation exhibited a significantly positive correlation with poor disease‐specific and metastasis‐free survival among breast cancer patients in clinics [139]. Collectively, suppressing AMPK may offer a potential effective onco‐therapeutic option.

Yet, AMPK was initially recognized as a tumor suppressor due to its inhibitory role in mTOR signaling that governs cell proliferation [140]. For instance, AMPK degradation led to promoted mTOR signaling and malignant transformation in lung [141], breast [142, 143], prostate [144], bladder [145], lung [146], gastric [147], and pancreas [148] tissues. AMPK depletion accelerated lymphomagenesis that was driven by *MYC* (a proteo‐oncogene that encodes a transcription factor involved in cell cycle, apoptosis, and transformation), and promoted the earlier onset of T‐cell lymphoma upon *p53* knockout [149]. The co‐deficiency of another tumor suppressor and early transforming stage of these studies suggested that activating AMPK may be appropriate to prevent pre‐malignant cells from developing into the cancerous state.

Given the oncogenic role of AMPK, there is a great need to develop therapeutic strategies suppressing AMPK. Dorsomorphin, though being a known AMPK inhibitor, could not be used for cancer treatment as it is highly toxic with off‐target effects [150]. On the other hand, several small molecules activating AMPK have been shown effective in reducing the tumorigenicity potential of cancer cells if used in combination with other anti‐cancer treatments. For example, combined treatment of prostate cancer cells using MT63‐78 (a small molecule of AMPK activator) and hormonal therapy inhibiting androgen receptor signaling resulted in a synergistic anti‐cancer effect [151]; canagliflozin in combination with ionizing radiation and the chemotherapy docetaxel activated AMPK and inhibited the tumorigenicity of prostate and lung cancer cells [152]; and AMPK activation by metformin in combination with CTLA4 (an inhibitory receptor for T cells) blockade enhanced the antitumor immunity among breast cancer cells [153]. In summary, AMPK inhibitors may be promising for cancer treatment by suppressing cancer survival, and AMPK agonists may be useful for cancer prevention and enhancing the efficacy of other onco‐therapeutic strategies via slowing down cell proliferation.

### 3.3 Dose‐dependent effect of free radicals on AMPK

Free radicals are known to be capable of modulating AMPK viaaltering key cysteine residues in the α1 subunit of the AMPK complex and affecting the mitochondrial respiratory enzymes [154, 155]. Free radicals were initially considered to increase AMPK activity by enhancing the AMP:ATP ratio, but later was found to cause extracellular regulated kinase (ERK1/2)‐mediated phosphorylation of the Thr172 residue in the catalytic α‐subunit of AMPK [154]. Yet, oxidation of other Cys residues (e.g., 130 and 174) in the α‐subunit of AMPK may induce AMPK aggregation and thus block it from being phosphorylated by its upstream kinases [155], implicating the critical importance of maintaining cellular redox homeostasis for activating and maintaining AMPK activity. They can be grouped into ROS and reactive nitrogen species (RNS), the effects of which on cells differ by the type and dosage (Figure 6).

**FIGURE 6:**
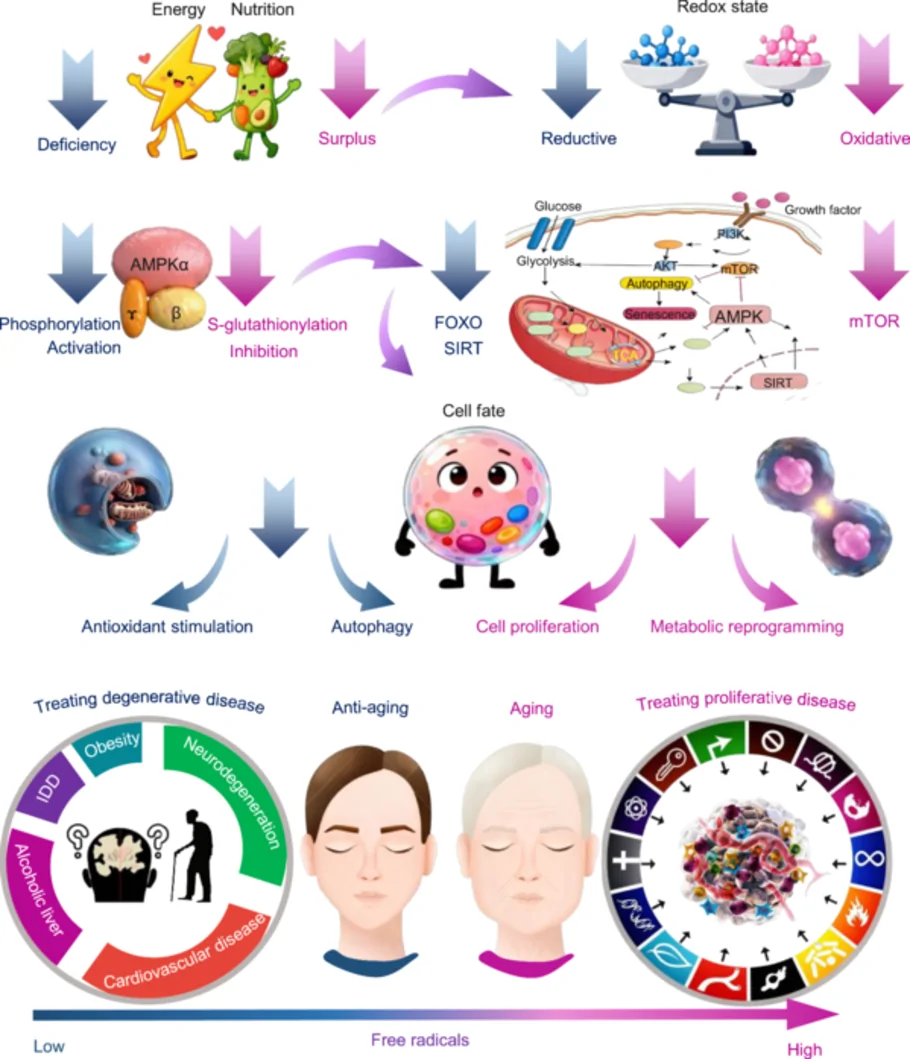
AMPK signaling integration map linking redox sensing to aging and disease management. This schematic diagram links upstream stimuli, cellular redox status, AMPK activity, and its major post‐translational modifications to downstream signaling axes, cell fates, and disease trajectories. In response to divergent energy and nutritional stimuli (i.e., deficiency vs. surplus) that shift the cellular redox balance (i.e., toward oxidative or reductive states), AMPK undergoes distinct primary post‐translational modifications (i.e., phosphorylation vs. inhibitory S‐glutathionylation) that determine its activation status. While activated AMPK promotes forkhead box O (FOXO) signaling and the NAD‐dependent deacetylase sirtuin (SIRT) axis, inhibited AMPK favors the mTOR pathway. This bifurcation results in opposing cell fate decisions. While the FOXO or the SIRT axis stimulates processes like autophagy and antioxidant machinery, the mTOR pathway promotes cell proliferation and metabolic reprogramming. These divergent cellular fates map onto physiological aging processes and pathological disease trajectories. Specifically, AMPK activation and its associated processes (i.e., antioxidant defense, autophagy) are implicated in protecting against degenerative diseases (e.g., neurodegenerative diseases, cardiovascular diseases, alcoholic liver disease, intervertebral disc degeneration, obesity) and promoting longevity, whereas sustained mTOR signaling and its outcomes (i.e., cell proliferation, metabolic reprogramming) are linked to accelerated aging and cancer progression. Beyond the dose required for metabolic reprogramming, excess free radicals become lethal to cancer cells, offering a strategic avenue for cancer treatment. The diagram thus encapsulates the central theme of redox hormesis and the dual, context‐dependent role of AMPK signaling in health and disease.

In general, low concentrations of ROS enhance AMPK activity via stimulating the anti‐oxidant machinery, and high ROS concentrations may down‐regulate AMPK activity via oxidation or post‐translational modifications such as S‐glutathionylation. For instance, treating cells with an appropriate dose of exogenous H_2_O_2_ and exposing cells to hypoxia for endogenous ROS production both enhanced AMPK activity in mouse fibroblast cells [156]; high ROS concentrations rapidly oxidized several AMPK cysteine residues, such as Cys299/304/312, leading to AMPK aggregation and inhibited phosphorylation in cardiomyocytes [155], and high glucose‐induced intense oxidative stress reduced AMPK activity in skeletal muscle cells both in vitro and in vivo [157].

RNS originate from the oxidation of NO that may represent important signaling entities at low levels but imposes cells with nitrosative stress when their concentration exceeds the physiological level [27]. While low levels of RNS lead to AMPK activation, high concentrations of RNS generate protein tyrosine nitration and S‐nitrosylation [27]. For example, exposing MCF7 breast cancer cells to exogenous NO resulted in the stimulation of AMPK and, consequently, increased autophagy [28]. Given the similarity between RNS and ROS on proteins and cell behaviors, it is rational to postulate the inhibitory role of RNS on AMPK activity at high concentrations that deserves intensive investigations.

It is worth mentioning that the effect of a specific RONS is so far poorly examined. Analyses of endogenous RONS are usually performed with reporter molecules that cannot discriminate among different redox agents, and of exogenous RONS largely rely on H_2_O_2_ and NO. While NO can be considered as the main component of RNS since it is the starting molecule for forming other RNS, H_2_O_2_ is insufficient to represent the effect of other ROS, especially those with short lifespans such as OH•, O_2_^•^^−^ and ^1^O_2_. With the rapid development of nanotechnologies, many types of RONS can be specifically detected. For example, intracellular O_2_^•^^−^ level can be examined using bioinspired copper single‐atom nanozyme (Cu‐SAzyme) [158] or porous cerium oxide nanoparticles (PN‐CeO_2_) [159]; and H_2_O_2_ can be measured using probes like HyPer and its variants such as HyPer‐7 [160]. Synthetic biology provides new approaches for detecting specific RONS species by constructing gene‐encoded fluorescent sensors for real‐time monitoring taking advantages of techniques such as optogenetics and gene editing. To determine the effect of endogenous ROS on AMPK activity under the physiological conditions, a study innovatively generated physiological ROS levels within mitochondria at complex I without disrupting OXPHOS using the mitochondria‐targeted redox cycler MitoParaquat (MitoPQ) [161, 162]. More intelligent approaches are thus needed to enable the examination of short‐lived RONS on AMPK and cell behaviors.

## 4 PRINCIPLES OF FREE RADICAL‐BASED APPROACHES FOR ANTI‐AGING

### 4.1 Anti‐oxidation for health maintenance

The rationale for using anti‐oxidative strategies to preserve youthful and rejuvenative features is to enhance the ability of non‐pathological human cells in neutralizing RONS for reduced cell damage when free radicals are overproduced or the ability of cells to maintain cell redox homeostasis is impaired [163, 164].

#### 4.1.1 Anti‐oxidant health supplements

Endogenous antioxidants can be enzymatic and non‐enzymatic. The primary antioxidant enzymes include, for example, SOD, catalase (CAT), and GPx. For instance, SOD can convert O_2_ to H_2_O_2_, which is further decomposed to water and O_2_ by CAT for reduced OH• production. Non‐enzymatic antioxidants such as vitamin E, bilirubin, and β‐carotene function by scavenging RONS and terminating the free radical chain reactions. Several anti‐oxidants have been commercialized as nutrient supplements to preserve youthfulness, with the dominant products being vitamins, polyphenols, and coenzyme Q10 (CoQ10).

Vitamins with anti‐oxidative roles include, for example, vitamins A, C, D, and E. Though a consensus exists on the use of vitamins for youth maintenance, the feasibility of applying vitamins for disease therapeutic intervention remains to be questioned. For instance, vitamins showed disappointing efficacy in reducing the mortality of several diseases and even, in some cases, significantly promoted the overall mortality [165]. The undesirable therapeutic efficacy of anti‐oxidants for disease control can be well explained by the proposed updated insights on the canonical free radical theory of aging. That is, though low level of free radical is beneficial for healthy cells to activate compensatory mechanisms and slow down aging, it is not useful and even accelerates disease progression by reducing the number of pathological cells undergoing programmed death. Yet, anti‐oxidants may be useful for disease control if being administrated at extremely high dosages, since that may reverse the redox level of pathological cells back to the normal state if no detrimental damage was made to DNA.

Polyphenols are secondary metabolites of plants, several members of which have been shown to delay the initiation of pathologies such as cancers, cardiovascular disorders, neurodegenerative conditions, and osteoporosis via attenuating inflammation [166]. As existing evidence only supports the use of polyphenols for reducing the disease incidence, anti‐oxidants may only be beneficial for healthy cells to maintain redox homeostasis but not advisable to treat pathological cells as a therapeutic intervention approach.

Different from vitamins and polyphenols, oral intake of CoQ10 provides significant symptomatic benefits for many diseases, including, for example, neurodegenerative disorders [167], diabetes [168], cardiovascular diseases [169], and cancers [170]. It is noteworthy that these diseases are associated with mitochondrial respiratory chain (MRC) disorder. Thus, the therapeutic efficacy of CoQ10 is attributable to its role in restoring the electron flow in the MRC, and CoQ10 supplementation may only be beneficial to patients carrying primary defects in the CoQ10 biosynthetic pathway.

#### 4.1.2 Ketogenic diet

The ketogenic diet, defined as high‐fat, low‐to‐moderate protein, and low‐carbohydrate diet (typically 3–4 g of fat to 1 g of carbohydrate and protein), makes cells depend on mitochondrial respiration for energy production much more than on glycolysis, leading to elevated ketone bodies and reduced blood glucose [171]. As ketone body metabolism is known to protect cells from oxidative damage by suppressing RONS synthesis and enhancing the endogenous antioxidant ability of cells, and glucose restriction is capable of increasing the response of normal cells to oxidative stress via enhancing CAT activity, ketogenic diet has been considered to be anti‐oxidative and beneficial for maintaining body fitness [172, 173, 174, 175].

Specifically, the ketogenic diet can activate NRF2 via redox signaling, leading to increased production of antioxidant glutathione (GSH) and an improved mitochondrial redox state [176, 177, 178, 179]. A study involving 20 healthy adult women investigated the effect of a ketogenic diet on the blood redox status, and the results showed that a short‐term (i.e., 14 days) ketogenic diet was associated with a significant weight loss and substantially improved blood antioxidative status [180].

#### 4.1.3 Moderate‐intensity non‐acute exercise

Physical exercise can be anti‐ or pro‐oxidative depending on its intensity and features. Moderate‐intensity exercise is a general recommendation of a healthy lifestyle [181,182], as either inactivity or high‐intensity physical exercise was associated with increased oxidative stress, and moderate‐intensity exercise was related to reduced redox levels, especially among people with advanced age [183]. The outcome of a particular physical exercise is also determined by its features, especially among elderly individuals. Specifically, acute exercise training was reported to elevate free radical production and activity of the antioxidant machinery, with the latter declining with age; resistance training improved the antioxidant defense; and endurance training both reduced the production of free radicals and increased the antioxidant defense among the elderly population [184]. Thus, a moderate‐intensity protocols of endurance or resistance training may be anti‐oxidative and recommended for healthy individuals, especially senior citizens. Accordingly, 50%–80% VO_2_max (defined as the maximum rate of O_2_ consumption measured during incremental exercise) for 2–3 sessions/week has been considered as optimal aerobic training intensity [183].

### 4.2 Anti‐oxidation for treating degenerative diseases

Alzheimer's disease, a type of neurodegenerative disease, is featured with the loss of neurons as a result of abnormal Aβ accumulation and/or hyperphosphorylation of tau proteins. With the advance of aging, the levels of free radicals such as H_2_O_2_, NO, HO• and O_2_^•^^−^ increase, leading to Aβ accumulation and the deposition of neurofibrillary tangles and, consequently, synaptic impairment and cell death according to evidence from various *in vitro* cell models, in vivo transgenic mice, and patients carrying Alzheimer's disease [185, 186, 187]. Several antioxidant strategies have been established for treating Alzheimer's syndrome. For instance, the combined use of vitamins C and E has been associated with reduced prevalence and disease activity among Alzheimer's patients [188, 189]. Also, the herbal medicine Ginkgo biloba has been proposed to be beneficial to treat Alzheimer′s carriers via activating antioxidant enzymes such as CAT, SOD, and heme oxygenase‐1 (HO‐1) [190, 191, 192].

Osteoporosis, being a type of spinal degenerative disease, is typically accompanied with bone loss. Osteoporosis predisposes patients to fracture, progressive spinal deformities, and stenosis [193]. Oxidative stress has been considered an important pathogenic factor of osteoporosis, which is positively correlated with the apoptosis of osteoblasts but negatively with that of osteoclasts [193]. Therapeutically, dietary intake of natural antioxidants has been suggested to be efficient in preventing and reducing the negative effects of oxidative stress on bone remodeling and osteocytes. Indeed, osteocytes have been considered potential biological targets of antioxidants for preventing and reducing bone loss, as well as promoting and restoring bone remodeling. Several data have indicated the effectiveness of using antioxidants in preventing and treating oxidative stress‐related bone diseases, including osteoporosis. For instance, low dose of vitamin C has been associated with high rates of bone turnover in a study using guinea pig as the animal model [194]; and a positive correlation between dietary vitamin C intake and bone mineral density among postmenopausal women has been established from the epidemiological investigation [195].

Metabolic syndromes such as type II diabetes, hypertension, and abdominal obesity are chronic non‐infective pathological states predisposing individuals with high risks of developing aging‐associated degenerative diseases. Type II diabetes, among other metabolic syndromes, is caused by a hostile metabolic environment that leads to β‐cell exhaustion and loss‐of‐function. As one of the leading causes of type II diabetes, hyperglycemia activated all pathways to break down the glucose, leading to increased production of free radicals such as HO•, O_2_^•^^−^, and H_2_O_2_ or impaired antioxidant defense by imposing pressure on the electron transport chain complexes and causing electron leakage at complexes I/II/III [196]. Antioxidant interventions have been considered capable of protecting β‐cells from oxidative stress‐induced apoptosis and thus diabetic‐related complications. For instance, increased levels of antioxidants such as vitamin D [197, 198], vitamin B6 [199], and vitamin E [200] were positively associated with improved β‐cell functionalities. At the epidemiological level, a strong link has been observed between the dietary antioxidant intake and protection against type II diabetes. For example, a randomized controlled study showed that delta‐tocotrienol (an antioxidant) supplementation significantly improved glycaemic parameters among individuals carrying diabetes [201].

### 4.3 Pro‐oxidation for treating proliferative diseases

The rationale for promoting the oxidative state of pathological cells with proliferative features for the reversal of detrimental and illness properties is to accelerate the death of pathological cells that typically possess higher levels of RONS than their healthy peers.

A growing body of evidence has indicated the essential role of antioxidants during tumorigenesis as they can rescue transformed cells from cell death induced by free radicals. Specifically, oncogenes such as *p21* and *MYC* have been shown capable of both inducing intracellular RONS [202, 203, 204, 205, 206] and stabilizing the anti‐oxidant protein NRF2 [207, 208, 209, 210, 211]; the expression of *NRF2* was found to be inducible by the breast cancer susceptibility 1 (*BRCA1*) gene, leading to improved cancer cell survival [212]. Additionally, high levels of FOXO family members (with known roles on antioxidation) have been implicated in cancers exhibiting high stemness, such as triple‐negative breast tumors [213]. Increased intracellular levels of GSH were required for the initiation and progression of various types of cancers [214]. Therefore, many existing onco‐therapeutic approaches turned out to be pro‐oxidative. For instance, therapeutics aiming at depleting GSH or blocking GSH synthesis such as sulfasalazine negatively affected cancer cell survival and appeared useful in treating bladder [215], esophageal [216], endometrial [217], colorectal [218], pancreatic [219] and triple negative breast cancer cells [220]; NOV‐002 was shown favorable in treating human epidermal growth factor receptor 2 negative (HER2‐) breast cancers by altering the ratio between oxidized GSH and GSH (GSSG/GSH) and inducing the oxidative stress [221].

On the other hand, the drastic use of anti‐oxidants may help control the aggressive properties of some cancers but cannot kill transformed cells. For example, a combination of nutritional antioxidants including 2850 mg vitamin C, 2500 iu vitamin E, 32.5 iu β‐carotene, 387 μg selenium plus secondary vitamins and minerals, essential fatty acids (1.2 g γ linolenic acid, 3.5 g n‐3 fatty acids), and 90 mg CoQ10 was administered to 32 advanced breast cancer patients aged 32–81 per day for 18 months, and the outcome was excellent. Specifically, no patient died during the investigation period, whereas four patients were expected to die if without receiving this treatment, no patient developed further distant metastasis, six patients showed apparent atrial remission, and the quality of life of all patients was improved [222]. Note that the efficacy of this regimen can be attributable to the high dosing of its antioxidants, as the recommended dietary allowances were 75–90 mg for vitamin C, was 22.35 iu for the natural form vitamin E, was 8.94–22.35 iu for the natural form β‐carotene, was 55 μg for selenium, and was 5 mg for CoQ10 for adults per day [223, 224].

## 5 CAP AS A PROMISING APPROACH FOR ANTI‐AGING

CAP, belonging to the fourth state of matter, is composed of a cocktail of RONS such as OH•, O_2_^•^^c^, ^1^O_2_, H_2_O_2_, O_3_, NO, and nitrite in the form of anion or proton (OONO^−^, ONOOH) [225]. The ample ingredients and large spectrum of free radicals, especially short‐lived ROS make CAP an ideal tool for exploring the effects of various sources of RONS on AMPK and cell behaviors, and investigations on the exact effect of each component possible, if coupled with scavengers of each type of free radicals. In addition, by varying parameters controlling the dosage of CAP such as the voltage, flow rate and treatment duration, one can easily tune the dosage of CAP generated for desirable cell outcomes; by choosing appropriate discharge mode such as dielectric barrier discharge, spark discharge and corona discharge, one can modulate the components of free radicals and their percentages to obtain the optimal treatment regimen (Table 1).

**TABLE 1:** Summary of CAP dosage and administration regimens of studies exemplified in this paper.

Device	Gas	Voltage	Frequency	Flow rate	Power	Exposure time	Distance to surface	Leading reactive species	Biological outcome	Model system	Ref
Jet (kINPen)	Ar	2–6 kV	1.7 MHz	5 L/min	3.5 W	10 min (10PAM)	10 mm	·OH, H2O2 (52.6 ± 1 mg/L), ·O2−, O3 (1.1 ± 0.1 mg/L), NO, NO3− (938.3 ± 15.4 mg/L), NO2− (22.7 ± 0.9 mg/L)	Cell death	Breast cancer cells	[61]
Jet (kINPen)	Ar	2–6 kV	1.7 MHz	5 L/min	3.5 W	10 min (10PAM)	10 mm	NA	Cell death	Breast cancer cells, bladder cancer cells	[226]
Jet	He	1–1.4 kV	8.8 kHz	1 L/min	NA	1–5 min	13 mm	·OH, H2O2, ·O2−, NO, O3	Cell death	Breast cancer cells	[214]
Jet	He	1–1.4 kV	8.8 kHz	1 L/min	NA	1–5 min	13 mm	NA	Cell death	Breast cancer cells	[227]
Jet	He	1.2 kV	8.8 kHz	1.2 L/min	NA	2–5 min	10 mm	NA	Cell death	Prostate cancer cells	[228]
Jet (P500‐SM)	Ar	8.5 kV	20 kHz	5 L/min	45 W	60 s	30 mm	NA	Cell death	Oral squamous cell cancer cells	[229]
Jet (invivoPen)	He	5 kV	8.8 kHz	0.2 L/min	NA	5 min	Inside tissue	NA	Tumor shrinkage	Breast cancer	[230]
Jet	90%He + 10%O2	10 kV	6 kHz	2 L/min	NA	60 s	10 mm	H2O2 (4–6 μM), NO2− (2–4 μM)	DNA damage	Esophageal cancer cells	[231]
Jet (Periplapy)	Ar	4.5 kV	NA	1.25 L/min ± 20%	NA	1 min	20 mm	O3 (0.008 ppm), NO (<0.001 ppm), NO2 (0.007 ppm)	Osteoblast proliferation & differentiation	Mouse embryo osteoblast precursor cells	[232]
Jet	Ar	3 kV	NA	1 L/min	3 W	0.5–2 min	10 mm	O3 (0.006 ppm)	Osteoblast differentiation	Periodontal ligament cell	[233]
Jet	Air	NA	NA	0.6 L/min	NA	60 s	70 mm	H2O2 (120 mM), NO2− (1.5 μM), NO3− (4 μM)	Macrophage polarization	Macrophages	[234]
Jet	Air	0.4 V	NA	150 L/h	NA	5–10 min	NA	NO2− (15–20 ppm), O3 (20–30 ppm)	Bacterial load reduction	*Escherichia coli, S. aureus, S. enterica, P. aeruginosa*	[235]
Jet	Air	>10 kV	30.5 MHz	NA	NA	9.5 min	NA	H2O2 (25 mg/L), NO2− (10 mg/L)	Bacterial load reduction	*Escherichia coli*	[236]
DBD	Ar	0–25 kV	9 kHz	2.5 L/min	NA	3 min	30 mm	NA	Cell death	Melanoma cells	[237]
DBD	Ar	29 kV	30 Hz	NA	NA	10 s	1 mm	NA	Cell death	Colorectal cancer cells	[238]
DBD	Ar	17 kV	500 Hz	NA	NA	10 s	1 mm	H2O2 (700 × 10^−6^ M), NO2− (1770 × 10^−6^ M), ONOO− (35 × 10^−6^ M)	Cell death	Melanoma cells	[239]
DBD (PlasmaONE®MEDICAL)	Air	3–18 kV	NA	NA	NA	1 min	NA	NA	Cell death	Osteoblast cells	[240]
DBD	Air, N2, O2	150 V	NA	2 L/min	0.9 W (Air), 1 W (N2), 0.9 W (O2)	1–3 min	15 mm	H2O2 (Air: 40–60 nM; N2: 18–35 nm; O2: 10–17 nm), NO (Air: 60–120 nM; N2: 25–60 nM; O2: 55–175 nM)	Neural maturation	Neural cells	[241]
DBD	He	6 kV	10 kHz	3 L/min	NA	5–10 s	16.2 mm	H2O2 (1.5–3 μM)	Osteoblast differentiation	Murine pre‐osteoblast cells	[242]
DBD	Ar	NA	60 Hz	1 L/min	2.4 W	30 s	NA	NA	Cell proliferation	Male chicken sertoli cells	[243]
DBD	He	NA	13.56 MHz	NA	100 W	15 s	NA	NA	Lifespan	*C. elegans*	[244]
	Ar	8 kV	36 kHz	1.5 L/min	NA	5–25 s	20 mm	NA	Collagen synthesis	Murine fibroblast cells	[245]
DBD	Air	8.3 ± 0.2 kV	24 ± 3 kHz	NA	NA	20–80 s	1.4 mm	NA	Transdermal topical lidocaine absorption	Mouse skin	[246]

*Note*: Device name is provided in brackets if it has. NA means “not available.”

Abbreviations: Ar, argon; DBD, dielectric barrier discharge; He, helium; Jet: plasma jet; PAM, plasma‐activated medium.

While numerous medical miracles have been demonstrated by CAP showcasing its feasibility in resolving medical issues such as wound healing [247], sterilization [248] and cancer treatment [61, 213, 249], novel efficacies of CAP indicating its innovative biomedical uses have been consecutively reported such as triggering the differentiation of neural stem cells [241] and osteoblasts [230–232] (Table 2). This, though thrilling us on what CAP can do, complicated its mechanism‐of‐action. Below, we summarize the potential roles of CAP for anti‐aging, the dosage of which is critical and requires standardization (Figure 7).

**TABLE 2:** Preclinical and clinical studies of CAP for medical applications exemplified in this paper.

Pathological condition	Study type	Sample size	Follow‐up time (in vivo/clinical)	Primary outcome	Ref
Advanced solid tumors	Clinics (NCT04267575)	20 patients	31 months	Safety, efficacy	[250]
Breast cancer	In vitro, in vivo	8 cell lines, 15 mice	19 days	Apoptosis	[61]
Breast cancer	In vitro, in vivo	7 cell lines, 50 mice	39 days	Cancer stemness	[213]
Breast cancer	In vitro, in vivo	4 cell lines, 6 mice	29 days	Apoptosis, tumor shrinkage	[249]
Breast cancer	In vivo	15 mice	30 days	Tumor shrinkage	[230]
Breast cancer	In vitro	4 cell lines	NA	Cancer stemness	[226]
Breast cancer	In vitro	5 cell lines	NA	EMT	[251]
Breast cancer	In vitro	6 cell lines	NA	Apoptosis	[252]
Breast cancer	In vitro	7 cell lines	NA	Viability	[238]
Bladder cancer	In vitro	2 cell lines	NA	EMT	[251]
Bladder cancer	In vivo	16 mice	40 days	Tumor shrinkage, survival	[253]
Colorectal cancer	In vitro, in vivo	1 cell line, 30 mice	28 days	ICD, tumor shrinkagte	[254]
Colorectal cancer	In vitro	1 cell line	NA	Invasion	[255]
Esophageal cancer	In vitro	2 cell lines	NA	DNA damage	[231]
Glioblastoma	In vitro, in vivo	1 cell line, 2 mice	5 days	Apoptosis, tumor shrinkage	[256]
Glioblastoma	In vitro	1 cell line	NA	Viability	[257]
Glioblastoma	In vitro	3 cell lines	NA	Sensitivity restoration	[258]
Lung cancer	In vitro	2 cell lines	NA	Apoptosis	[253]
Lung cancer	In vitro	1 cell line	NA	Apoptosis	[259]
Melanoma	In vitro, in vivo	2 cell lines, 32 mice	50 days	ICD	[239]
Melanoma	In vitro	3 cell lines (2 cancer, 1 macrophage)	NA	Macrophage polarization	[234]
Melanoma	In vitro	2 cell lines	NA	Apoptosis, macromolecular structure	[237]
Melanoma	In vitro	2 cell lines	NA	Apoptosis	[259]
Melanoma	In vitro	1 cell line	NA	Apoptosis	[253]
Melanoma	In vitro	1 cell line	NA	Apoptosis	[260]
Oral squamous cell cancer	In vitro	2 cell lines	NA	Drug sensitivity	[229]
Osteosarcoma	In vitro	3 cell lines	NA	Apoptosis	[259]
Pancreatic cancer	In vitro, in vivo	4 cell lines, 10 mice	29 days	Apoptosis, tumor shrinkage	[261]
Pancreatic cancer	In vitro, in vivo	1 cell line, 36 mice	48 days	Drug sensitivity, tumor shrinkage	[262]
Pancreatic cancer	In vitro, in vivo	1 cell line, 40 mice	70 days	Metabolism, proliferation, apoptosis	[263]
Pancreatic cancer	In vitro, in ovo	4 cell lines, 18 fertilized chicken eggs	6 days	Metastasis	[264]
Pancreatic cancer	In vitro	1 cell line	NA	Viability, death	[265]
Pancreatic cancer	In vitro	1 cell line	NA	Viability	[266]
Pancreatic cancer	In vitro	4 cell lines	NA	ICD	[267]
Pancreatic cancer	In vitro	2 cell lines	NA	Viability	[268]
Pancreatic cancer	In vitro	3 cell lines	NA	Apoptosis	[269]
Prostate cancer	In vitro	2 cell lines	NA	G_0_/G_1_ arrest, apoptosis	[228]
Anti‐aging	In vitro, in vivo	1 cell line, 1 *C. elegans* model	30 days	Lifespan	[244]
Fibroblast collagen synthesis	In vitro	1 cell line	NA	Viability, collagen synthesis	[245]
Neural differentiation	In vitro, in ovo	1 cell line, 1 zebrafish embryo	24 h	Neural maturation	[241]
Osteoblast proliferation	In vitro	1 cell line	NA	Apoptotic markers	[240]
Osteoblast differentiation	In vitro	1 cell line	NA	Proliferation, ALP activity, calcium deposition	[233]
Osteoblast differentiation	In vitro	1 cell line	NA	Proliferation, ALP activity, calcium deposition	[242]
Osteoblast proliferation & differentiation	In vitro	1 cell line	NA	Proliferation, ALP activity, calcium deposition	[232]
Sertoli cell proliferation	In vitro	Male chicken Sertoli cells	NA	Viability, growth, cell cycle, mitochondrial activity	[243]
Transdermal delivery	Ex vivo	30 mice	NA	Transdermal topical lidocaine absorption	[246]
Bacteria	In vitro	4 bacteria	NA	Inactivation	[235]
Bacteria	In vitro	1 bacteria	12 h	Inactivation	[236]

*Note*: NA means “not available.” Follow‐up time is only recorded for in vivo, in ovo, or clinical studies.

Abbreviations: ALP, alkaline phosphatase; EMT, epithelial‐mesenchymal transition; ICD, immunogenic cell death.

**FIGURE 7:**
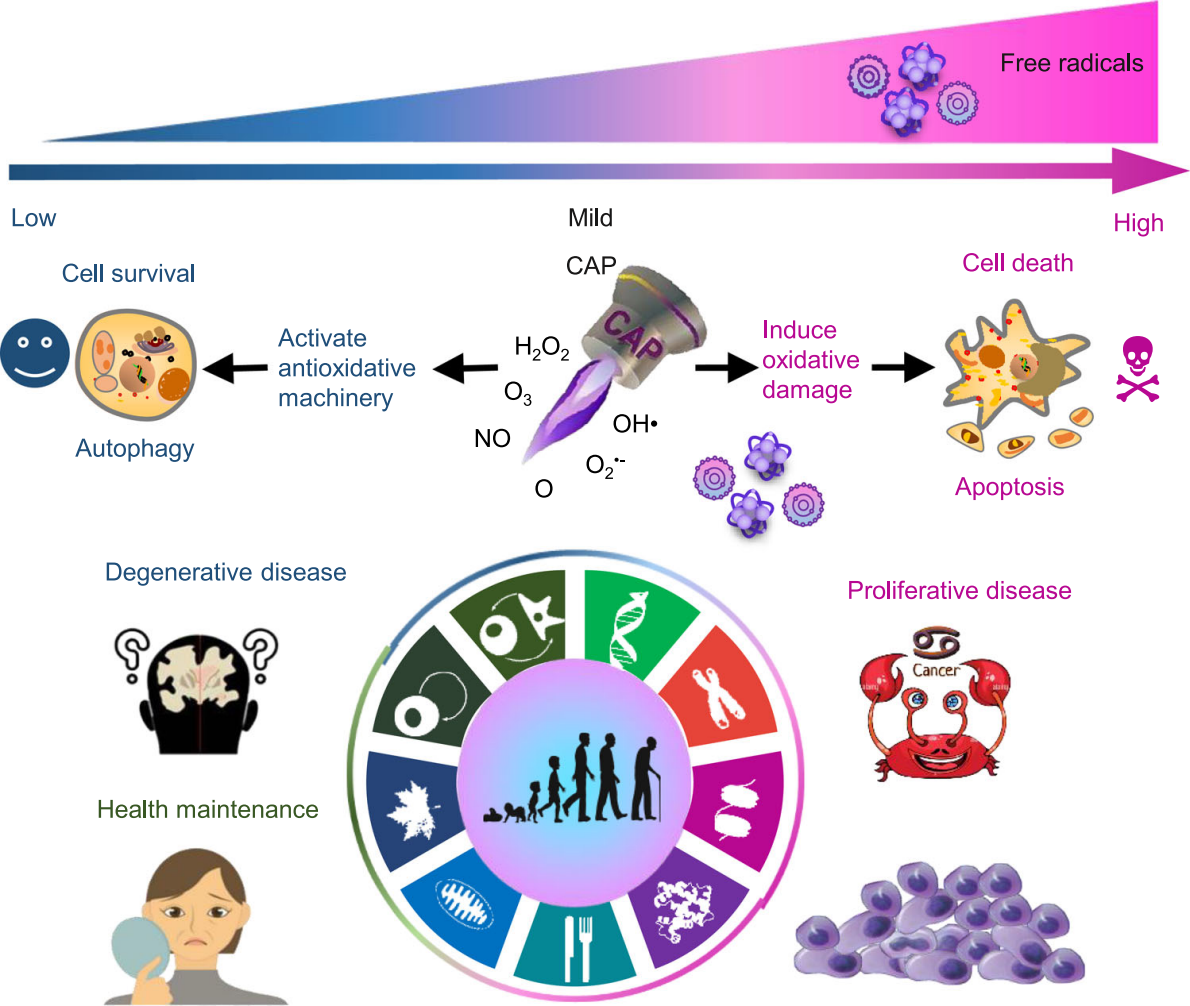
Simplified illustration on the specification of CAP as a free radical‐based approach for anti‐aging. Cold atmospheric plasma (CAP), composed of a cocktail of free radicals, can activate the antioxidant machinery of cells for survival via inducing, for example, autophagy, and induce oxidative damage to cells for death via triggering, for example, apoptosis. Age‐associated pathological conditions can be simplified into degenerative and proliferative diseases, where mitochondrial syndromes are combined with degenerative disorders due to the predisposing role of metabolic disorders on degenerative diseases. Low‐to‐mild dose of CAP can be used for health maintenance and treating degenerative diseases via enhancing cell survival, and mild‐to‐high dose of CAP can be used for treating proliferative diseases, as exemplified by cancers. Note that more complicated and multiple mechanisms may coexist in a particular case due to the complexity of the disease pathogenesis and the heterogeneity of the body environment.

### 5.1 Low‐to‐mild dose of CAP for health maintenance

Though relatively little has been investigated on the use of CAP for rejuvenation and keeping the fitness, a study employing *Caenorhabditis elegans* as the modeling organism was conducted to examine the potential of CAP for anti‐aging. The results showed that both the lifespan and the movement behavior of nematodes were significantly improved after being treated with CAP at a moderate energy density [244]. Besides, several studies have reported the potential use of CAP in resolving infertility. Specifically, CAP, generated at low physiological levels, was shown to be capable of promoting the growth, migration, and differentiation of Sertoli cells (SCs) via activating AMPK signaling, suggesting an innovative application of CAP for improving the quality of sperms [243] (Table 2). Another interesting study reported the dose‐dependent property of CAP in boosting collagen synthesis for skin rejuvenation. Specifically, an argon CAP jet was employed to treat L929 murine fibroblasts cultured in vitro, and the viability and collagen synthesis ability of fibroblasts were both significantly enhanced in response to CAP treatment for 15 s; yet this effect was highly dose‐dependent as remarkably decreased fibroblast viability and collagen level were observed when the treatment duration was increased to 25 s [245] (Table 2). This study attributed enhanced collagen synthesis to CAP‐induced cell cycle arrest [245]. Being both critical events regulating cell proliferation, whether AMPK‐related autophagy is also involved in driving this phenomenon deserves further investigations.

Given that the cellular outcome of CAP is highly dose‐dependent, it requires special cautions to carefully control the treatment duration and frequency for the purpose of anti‐aging. Thus, large‐scale clinical investigations are needed to determine these parameters as well as the associated demographic features before CAP can be truly applied as a rejuvenation tool. It may also help if representative clinical manifestations post‐CAP exposure could be identified from these clinical data, which can help users dynamically adjust the treatment dosage based on symptoms they display after receiving CAP treatment. It is worth mentioning that tumor patients or people who are subjected to high risks of developing tumors should avoid using CAP for anti‐aging that may stimulate tumor onset or progression, provided with its seemingly paradoxical roles under different dosages.

### 5.2 Low‐to‐mild dose of CAP for treating degenerative diseases

Low‐to‐moderate levels of oxidative stress can stimulate AMPK for activating the anti‐oxidant ability in cells [270], and differentiated cells exhibit higher oxidative levels than undifferentiated cells in general [271]. It is thus possible to treat degenerative diseases by applying low doses of CAP. That is, by slightly enhancing the redox level of stem cells without exceeding the tolerable physiological level, it is possible to treat degenerative diseases by promoting the differentiation of the corresponding progenitor cells; and by mildly enhancing the redox stress of degenerative cells, it is possible to rescue their impaired antioxidant machinery.

Indeed, an appropriate dosage of CAP has been shown to becapable of enhancing the differentiation of neural stem cells [241] and osteoblasts [242] (Table 2), and thus been proposed feasible for treating neurodegenerative diseases such as Parkinson's and Alzheimer's diseases as well as spinal degenerative diseases such as osteoporosis and IDD.

### 5.3 Mild‐to‐high dose of CAP for treating proliferative diseases

As AMPK has been attributed to aberrant mitochondrial dynamics and cell metabolism, targeting AMPK may open up a new avenue for cancer therapies [133]. In general, high concentrations of CAP may be feasible for treating cancers by suppressing AMPK activity for ablated tumor cell survival. Yet, activating AMPK via suppressing mTOR signaling may also be beneficial for cancer control if coupled with other onco‐therapeutics [272, 273, 274].

Indeed, CAP has demonstrated its unique specificity against a plethora of malignant cells such as melanoma [234, 237, 275], breast cancer [61, 213, 226, 249], prostate cancer [228], bladder cancer [226], pancreatic cancer [276], esophageal cancer [231], oral squamous cell cancer [229], and glioblastoma [231], without causing obvious harm to their healthy peers. This may, at least partially, be attributed to the differential sensitivities of AMPK to redox stimulus between healthy and transformed cells, as well as different cell outcomes in response to AMPK activation, which deserve further experimental validations. As the molecular outcome of cancer cells to CAP treatment, CAP has been shown to be capable of triggering various death programs [61, 239], suppressing migration [226] and targeting cancer cell stemness [213] ever since 2007 when its anti‐cancer efficacy was firstly reported. In clinics, CAP has been used to rescue the life of a 75‐year‐old advanced pancreatic cancer patient in 2016, and secure the life of a 33‐year‐old rare relapsed peritoneal sarcoma patient in 2019 [277]. In the first clinical trial examining the efficacy and safety of CAP as an oncotherapy (NCT04267575) that was initiated in 30 July 2019, 17 out of 20 late‐stage solid cancer patients (diagnosed with 2–3 months lifetime onset) were still alive by the end of the trial in 14 April 2021 [250], suggesting the feasibility of using CAP as an innovative oncotherapy (Table 2).

Though intensive efforts have been devoted to decipher the mechanism driving the anti‐cancer efficacy of CAP, little focus has been laid on the functionality of AMPK signaling in this process that may underlie the shared evolutionary path between aging and carcinogenesis. This makes the identification of critical metabolic transition points bridging the transformation from aging‐related physiological syndromes to pathological disorders relevant and urgent.

## 6 CONCLUSIONS

The canonical free radical theory of aging emphasized the essential role of oxidative damage accumulation caused by free radicals in aging. Incremental evidence has challenged the general validity of this theory, with multiple adapted versions being proposed to substantiate its content. Typical models include the “polymerase γ theory of aging” and the “spontaneous decay model” that, although both relating the aging process to mtDNA transition mutation burdens, differ in attributing the cause of mtDNA transition to accumulated mtDNA replication errors (the “polymerase γ theory”) and spontaneous mtDNA decay (the “spontaneous decay model”), respectively. Paradoxically, there also exists plenty of evidence supporting the beneficial role of free radicals in maintaining the normal functionalities of mitochondria if under appropriate control, leading to the concept of mitohormesis.

Though a single piece of evidence or experiment that does not fit in a theory is sufficient to disprove its general validity in principle, evidence challenging the integrity of the canonical free radical theory of aging does not mean that all experiments in favor of this canonical theory are invalid or of no value in practice; instead, these facts implicate that the traditional belief is lopsided and requires substantiation or adaptation. This has already fostered plenty of studies and will attract more researchers towards its improvement. With a great interest, we endeavored to consummate this canonical free radical theory of aging by emphasizing the dose‐dependent dual roles of reactive species in human health (with specific recommendations on free radical dosage being provided for each type of health concerns), characterized AMPK as the nexus between mitochondria health and free radicals as well as, importantly, potentiated the use of CAP for health management once under appropriate dosage control.

Rapidly evolving multi‐omics technologies can refine the “free radicals‐mtDNA‐AMPK‐aging” model and the validity of CAP for its proper intervention, providing a powerful forward‐looking perspective. Existing genomics and transcriptomics datasets could be re‐analyzed to scrutinize this framework by examining age‐stratified patterns of mtDNA mutations and deletions, testing if their accumulation was correlated with a transcriptional downregulation of nuclear‐encoded mitochondrial genes and the onset of oxidative phosphorylation dysfunction [278]. Furthermore, single‐cell RNA sequencing of aged tissues such as skin has revealed cell‐type‐specific transcriptional programs and increased stress response regulons, offering a high‐resolution view into how mitochondrial stress signatures and context‐dependent AMPK pathway activity vary across different cell populations during aging [279]. Proteomic studies may add another critical layer of validation, as organ‐specific and plasma proteomic aging clocks have identified synaptic loss, vascular dysfunction, and inflammatory pathways as key age‐related processes [280]. Crucially, proteomic analyses of biofluids from cognitively normal individuals across ages detected increased inflammatory mediators and extracellular matrix components, alongside specific peptide‐level modifications, providing a molecular readout of the systemic consequences of aging that could be linked to declining AMPK activity and compromised mitochondrial fidelity [281]. Metabolomic atlases further grounded the model in measurable biochemical shifts, revealing the strong predictive roles of metabolites like alpha‐ketoglutarate and hydroxyproline on organ age, thereby directly connecting metabolic homeostasis, a core output of AMPK signaling, to the aging process [282]. Finally, integrated transcriptomic and metabolomic analyses in intervention studies, such as those investigating anti‐aging peptides, demonstrated the capacity of multi‐omics in revealing coordinated changes in central metabolic pathways like the TCA cycle and glutamatergic signaling, effectively showcasing how these approaches could map the network‐wide effects of perturbing the proposed model's components [283]. Importantly, CAPmed‐BC, the first comprehensive data repository systematically capturing breast cancer cells' response to CAP intervention through integrated multi‐omics profiling, has been established, unifying molecular measurements across whole transcriptomics, proteomics, phosphorylomics, acetylomics, and lactylomics, thereby offering unprecedented insights into context‐dependent transcriptional and post‐translational regulatory dynamics that can directly validate and refine the proposed “free radicals‐mtDNA‐AMPK‐aging” framework [284]. Together, these diverse omics studies have not only resonated with the model′s predictions but also provided a rich, data‐driven framework for its iterative refinement by pinpointing specific molecular interactions, compensatory mechanisms, and tissue‐specific vulnerabilities across the lifespan.

CAP exhibits remarkable versatility in clinical applications, enabling diverse delivery modalities tailored to specific therapeutic needs. It can be administered via direct exposure using plasma sources such as dielectric barrier discharge (DBD) [285, 286, 287] or indirect‐discharge systems like plasma jets and plasma torches [288]. While direct CAP exposure has been well‐established with several of which are already being commercialized, including MicroPlaSter, plasma care®, MIRARI® Cold Plasma System, PlasmaDerm® VU‐2010, kINPen®MED and SteriPlas [288], its limited penetration in‐depth, that is, approximately 2 mm, has largely restricted its application to superficial regions. To overcome this, devices such as the *invivo*Pen have been developed for intradermal plasma ejection, enabling direct CAP delivery into tissues [230]. More commonly utilized are CAP‐activated media including Ringer's lactate solution [246, 289], phosphate‐buffered saline [267], and hydrogels [290], which can be topically applied [291] or administered via subcutaneous injection [265]. Transdermal delivery has been further enhanced through nanotechnology‐based strategies such as microneedle systems [246, 289]. Additionally, CAP‐activated aerosols [235, 236] offer access to anatomically challenging or internal sites such as the respiratory tract, expanding applicability to minimally invasive surgical contexts. This broad spectrum of delivery forms of CAP has significantly augmented its potential to address a wide range of clinical challenges including anti‐aging.

If life could be portrayed as a metabolic procedure, aging is a process gradually losing the redox homeostasis that is manifested as various age‐related physiological symptoms and pathological syndromes. While food, water and oxygen (which are in the form of solid, water and gas, respectively) are indispensable materials driving the life process, CAP (which belongs to the fourth state of matter) may be the existing natural power to rectify the redox disequilibrium created during this progress. AMPK, being a nutrient and energy sensor, dictates the response of cells to external and internal stimuli, including free radicals from CAP for maintaining cellular redox homeostasis of cells. Thus, CAP may represent an ideal tool for investigating the efficacy of free radicals on human health and an innovative therapeutic approach for anti‐aging, alone or in combination with other interventions, at appropriate use of dosage. Investigating how AMPK in normal cells at different chronological stages respond to CAP of different dosages may represent one simple way at hand to help open our horizon on how free radicals can be harnessed for the benefits of human health including anti‐aging.

## AUTHOR CONTRIBUTIONS

**Xiaofeng Dai:** conceptualization, writing – original draft preparation. **Ruohan Lyu:** visualization. **Meilan Hu:** funding acquisition. All authors have read the final manuscript and approved it for publication.

## CONFLICT OF INTEREST STATEMENT

The authors declare no conflicts of interest.

## ETHICS STATEMENT

No animals or humans were involved in this study.

## Data Availability

No new data and scripts were used for this review. Supplementary materials (graphical abstract, slides, videos, Chinese translated version, and update materials) may be found in the online DOI or iMetaOmics http://www.imeta.science/imetaomics/.
